# Phenotypic Plasticity of the Mimetic Swallowtail Butterfly *Papilio polytes*: Color Pattern Modifications and Their Implications in Mimicry Evolution

**DOI:** 10.3390/insects13070649

**Published:** 2022-07-19

**Authors:** Tomoyuki Shimajiri, Joji M. Otaki

**Affiliations:** The BCPH Unit of Molecular Physiology, Department of Chemistry, Biology and Marine Science, Faculty of Science, University of the Ryukyus, Okinawa 903-0213, Japan; shimajiri@bcph.sci.u-ryukyu.ac.jp

**Keywords:** mimicry, the common Mormon butterfly, phenotypic plasticity, color pattern, *Papilio polytes*, sodium tungstate, temperature shock

## Abstract

**Simple Summary:**

Diverse butterfly wing color patterns are evolutionary products in response to environmental changes in the past. Environmental stress, such as temperature shock, is known to induce color pattern modifications in various butterfly species, and this phenotypic plasticity plays an important role in color pattern evolution. However, the potential contributions of phenotypic plasticity to mimicry evolution have not been evaluated. Here, we focused on the swallowtail butterfly *Papilio polytes*, which has nonmimetic and mimetic forms in females, to examine its plastic phenotypes. Cold shock and heat shock treatments in the nonmimetic form induced color pattern modifications that were partly similar to those of the mimetic form, and nonmimetic females were more sensitive than males and mimetic females. These results suggest that phenotypic plasticity in nonmimetic females might have provided a basis of natural selection for mimetic color patterns during evolution.

**Abstract:**

Butterfly wing color patterns are sensitive to environmental stress, such as temperature shock, and this phenotypic plasticity plays an important role in color pattern evolution. However, the potential contributions of phenotypic plasticity to mimicry evolution have not been evaluated. Here, we focused on the swallowtail butterfly *Papilio polytes*, which has nonmimetic and mimetic forms in females, to examine its plastic phenotypes. In the nonmimetic form, medial white spots and submarginal reddish spots in the ventral hindwings were enlarged by cold shock but were mostly reduced in size by heat shock. These temperature-shock-induced color pattern modifications were partly similar to mimetic color patterns, and nonmimetic females were more sensitive than males and mimetic females. Unexpectedly, injection of tungstate, a known modification inducer in nymphalid and lycaenid butterflies, did not induce any modification, but fluorescent brightener 28, another inducer discovered recently, induced unique modifications. These results suggest that phenotypic plasticity in nonmimetic females might have provided a basis of natural selection for mimetic color patterns during evolution.

## 1. Introduction

The phenotypes of organisms are determined by genotypes and environmental factors. Most, if not all, organisms show phenotypic plasticity in response to environmental stress during development. The contributions of phenotypic plasticity to natural selection, genetic assimilation, and speciation have been an active research area in ecology, evolutionary biology, and developmental biology [1,2,3,4,5,6,7,8]. There are at least two different types of phenotypic plasticity, “developmental conversion” and “phenotypic modulation” (sensu Smith-Gill (1983) [8]), although their distinctions may not always be clear [7,8].

Representative cases of developmental conversion are found in many insects, such as social castes in bees, ants, and termites, solitarious and gregarious forms of locusts, large and small horns with various shapes in beetles, winged and wingless forms in aphids, and seasonal color pattern polyphenism in butterflies [6,7,9]. In some butterflies, such as *Araschnia levana* in Europe and Asia, *Precis octavia* in Africa, and *Junonia almana* in Southeast Asia, wing color patterns differ drastically in different seasons [9]. The seasonal polyphenism in butterflies is controlled by ecdysteroid hormones [9,10,11,12]. Developmental conversion is understood as the expression of genetically determined alternative pathways in response to environmental cues such as temperature and light conditions. This type of plasticity is supposed to be adaptive as an evolutionary consequence. Similarly, there are numerous cases of genetically determined polymorphism of color patterns in butterflies, among which sexual dimorphism is well-known [13,14]. Furthermore, females often have two or multiple forms within a species. Such phenotypic variation within a species is supposed to be adaptive and is often controlled by regulatory genes that are not programmed to respond to environmental cues. A good example is a mimetic swallowtail, the common Mormon butterfly *Papilio polytes*, distributed widely in Southeast Asia [15,16,17].

Butterflies also show phenotypic modulation, which is the topic of the present study. This phenotypic plasticity is not adaptive at least at first and is likely an expression of a system failure during development in response to severe environmental conditions. In other words, this phenotypic plasticity was not built in as alternative genetic pathways. Nonetheless, this system failure is mild and completely viable. In butterflies, phenotypic modulation is known to cause two types of color pattern modifications: modifications induced by temperature shock and those induced via the general stress response [18,19,20]. Studies of color pattern modifications induced by temperature shock from evolutionary and developmental viewpoints were pioneered by Shapiro (1980, 1981) [21,22] and Nijhout (1984) [9,23]. Subsequently, Otaki (1998) [24] discovered that similar, if not identical, color pattern modifications can be induced by injections of oxyanions (such as sodium tungstate) into pupae. Other modification inducers, such as acid carboxypeptidase [25] and heparin and other sulfated polysaccharides [26], were also discovered later. These modification inducers or temperature shock treatments have been used for many lepidopteran species to understand developmental physiological aspects of color pattern formation [27,28,29,30,31,32,33]. These temperature-shock-type (TS-type) modifications appear to be induced via a systemic factor (i.e., a cold shock hormone) [24,34] as well as a local factor (i.e., an extracellular matrix) [24,35,36].

Importantly, color patterns of TS-type modifications of a given species are often similar to natural color patterns of different but related species in Nymphalidae, as discussed in *Nymphalis* [21,22] and *Junonia* [9,37]. The evolution of *Vanessa* (sensu stricto) nicely parallels the degrees of color pattern “modifications”, suggesting that environmental temperature shock might have played an important role in color pattern evolution and speciation in this genus [38,39,40]. Color pattern modifications induced via the general stress response also appear to have contributed to the evolution of *Vanessa* (*Cynthia*) *kershawi* [41]. Lycaenid butterflies are also modified [42], and color pattern modifications in the pale grass blue butterfly in the field may be considered a real-time evolution of color patterns [43] and may be one of the best cases of genetic assimilation in the field [5]. A similar case is also found in the pierid butterfly *Colias erate* [27]. These nymphalid, lycaenid, and pierid cases of phenotypic plasticity have provided evidence for the contribution of phenotypic plasticity to the color pattern evolution of butterflies. However, to our knowledge, no papilionid cases of phenotypic plasticity that might have contributed to color pattern evolution have been reported, and potential contributions of phenotypic plasticity to the evolution of dimorphic or multimorphic color patterns within a species, such as mimetic and nonmimetic forms of *P. polytes*, have not yet been evaluated.

**Figure 1:**
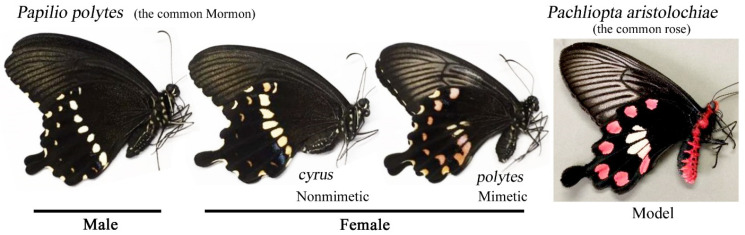
Nonmimetic and mimetic color patterns of the common Mormon butterfly *Papilio polytes* and its model, the common rose butterfly *Pachilopta aristolochiae*. The female color patterns of *P. polytes* are dimorphic in Okinawa, Japan: the nonmimetic *cyrus* form and the mimetic *polytes* form.

The female-limited mimicry not only in *P. polytes* but also in many butterflies has been explained by the high cost of expressing mimetic color patterns that can compensate for predation, which is not affordable for males [44,45,46,47,48,49]. While this line of explanation is reasonable, it does not take phenotypic plasticity into account. Similarly, although genes involved in the mimetic phenotype in *P. polytes* have been identified as *doublesex* (*dsx*) [50,51,52,53,54] and other genes, such as *cortex* [55], the question of how mimicry evolved in the first place requires multiple approaches, including studies on phenotypic plasticity. Here, we focused on the mimetic swallowtail butterfly, the common Mormon, *P. polytes*, in Okinawa, Japan [15,16,17], to examine its phenotypic plasticity. In Okinawa, this butterfly mimics the unpalatable swallowtail butterfly *Pachliopta aristolochiae*, distributed sympatrically (Figure 1). While males of *P. polytes* have a single nonmimetic form, females have a mimetic form (forma *polytes*) and a nonmimetic form (forma *cyrus*) (Figure 1). In the present study, we obtained color pattern modifications in this butterfly induced by cold shock or heat shock. Additionally, we injected a known modification inducer, sodium tungstate [24], and a recently discovered inducer, fluorescent brightener 28 (FB28) [56]. The induced modifications were compared to the mimetic color patterns, and we discussed the possible origin of the mimetic color patterns in this butterfly from the viewpoint of phenotypic plasticity.

## 2. Materials and Methods

### 2.1. Nomenclature of Spots and Key Differences between Nonmimetic and Mimetic Forms

**Figure 2:**
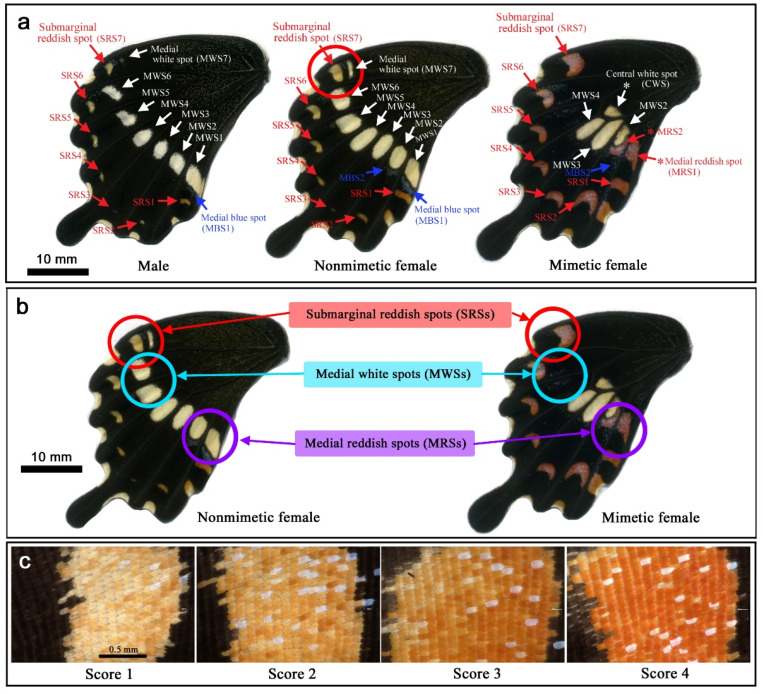
Color patterns among males, nonmimetic females, and mimetic females in *P. polytes* used in this study. (**a**) Nomenclature of color pattern spots. Asterisks indicate spots unique to mimetic females. The red circle indicates SRS7, which was examined for the darkness of reddish color. (**b**) Three main differences in color pattern spots between nonmimetic and mimetic females. In addition to SRSs, MWSs, and MRSs, CWS is unique to the mimetic form (not indicated in this panel). (**c**) Reddish spot scores for modifications. The most anterior submarginal reddish spot (SRS7) was used to assign scores visually in accordance with these images.

We focused on the ventral hindwings of *P. polytes* in this study because it was in the ventral hindwings that color pattern modifications were mainly observed. An exception is the ventral forewings, which showed an increase in sparse yellow scales. The nomenclature of the spots in the ventral hindwings in the present study is shown in Figure 2a. Seven submarginal reddish spots (SRS1–SRS7) and seven medial white spots (MWS1–MWS7) together with one or two medial blue spots (MBS1–MBS2) are the main color patterns of males and nonmimetic females. Marginal white spots (occasionally slightly reddish) were not examined. The SRSs are often simply called the “red spots” in other studies, but their color is closer to reddish brown and is clearly different from that of the “red spots” in the model species *P. aristolochiae*. Indeed, pigments in these two species are chemically different [57]. Therefore, these submarginal spots in *P. polytes* are called the “reddish spots” in this paper. The MBSs show structural color and may be sparse patterns [9]. In mimetic females, seven SRSs are maintained but are larger in size and darker in color than those in nonmimetic females. In contrast, the MWSs 1, 5, 6, and 7 are absent in mimetic females. MBS1 is also absent. Additionally, two medial reddish spots (MRS1 and MRS2) and the central white spot (CWS) are present only in mimetic females.

Key differences between the nonmimetic and mimetic forms are summarized in Figure 2b. There are three points. First, the SRSs, especially the most anterior one, are different in size and darkness: they are smaller in size and lighter in color in the nonmimetic form than in the mimetic form. Second, the MWSs are different: anterior ones exist in the nonmimetic form but do not exist in the mimetic form. Third, the MRSs are different: they do not exist in the nonmimetic form and exist in the mimetic form. The white spots near the MRSs are compromised in the mimetic form.

In this and many other papilionid butterflies, the background is dark-colored, and spots are light-colored. This coloration seems to be inconsistent with the binary color rule [30,31,58]. The binary color rule posits that color pattern elements are expressed in dark colors, and the background is expressed in light colors. Mechanistically, the induction model for color pattern determination states that dark (activator) signals always accompany light (inhibitor) signals and that light signals may behave independently [58,59,60]. This issue can be resolved by introducing a reversible positive–negative relationship, but the detailed explanations await future studies. Therefore, at present, we did not call these spots in *P. polytes* elements.

### 2.2. Butterflies and the Host Plant Leaves

Nonmimetic and mimetic females of *P. polytes* were caught in Shiohira (Itoman City), Maesato (Itoman City), Yamashiro (Itoman City), and Senbaru (Nishihara Town), Okinawa Prefecture, for egg collection in the laboratory from May 2020 to October 2021. These localities are all located in the southern region of Okinawa-jima Island. No clear difference in wing color patterns was observed among these females except for the nonmimetic and mimetic forms. Only individuals with low levels of wing damage were used to collect approximately 100 eggs per female individual. Egg collection was started on the same day as collection in the field.

One of the host plants of this butterfly in Okinawa, *Toddalia asiatica*, was exclusively used for egg collection and rearing. This plant is a small bush tree often found in the seacoast. Leaves were collected from various sites in Itoman City and from the Nishihara Campus of the University of the Ryukyus. When this plant was found in a city park or in a private land, permissions for leaf collection were obtained from Itoman City or from the personal owner of the land.

### 2.3. Sibling Groups

In this study, we set up sibling groups (SGs) based on mother butterflies, SG1 to SG8, in accordance with the experimental order of time (Appendix A, Table A1). Two groups, SG2 and SG6, were obtained from mimetic mothers, and other groups were obtained from nonmimetic mothers. For pilot experiments, a few nonmimetic mothers other than SG1–SG8 were used for egg collection. Mimetic mothers produced both mimetic and nonmimetic offspring individuals. Nonmimetic mothers produced either only nonmimetic offspring individuals or both mimetic and nonmimetic offspring individuals.

### 2.4. Egg Collection and Larval Rearing

For egg collection, a female was confined in a glass tank (300 mm × 300 mm × 300 mm) together with a bunch of the host plant collected from the University campus under the conditions of L16:D8 at 26 ± 2 °C in the laboratory. Twofold-diluted POCARI SWEAT (Otsuka Pharmaceutical, Tokyo, Japan) was administered to adults every day. A plant bunch was replaced with a new bunch before withering. The plant with deposited eggs was transferred to a plastic container (168 mm × 168 mm × 57 mm). Depending on the larval body size, we adjusted the number of larvae per container: 20 larvae or more for the first and second instar larvae, five to ten individuals for the third and fourth instar larvae, and just a single individual for the last instar larvae. Larvae were reared under the conditions of L16:D8 at 26 ± 2 °C. The host plant leaves were washed before use, and the leaves given to larvae were replaced with fresh leaves every day. When pupated, the pupae were randomly allocated to either the experimental (treatment) or control (no treatment) group. Each individual was determined to be a male, a nonmimetic female, or a mimetic female after eclosion.

### 2.5. Cold Shock and Heat Shock Treatments

For cold shock, within 12 h post-pupation, the pupae were transferred to an incubator set at −4.0 °C or −6.0 °C for 24 h or 48 h in a series of pilot experiments. Similarly, for heat shock, within 12 h post-pupation, the pupae were transferred to an incubator set at 38 °C, 39 °C, 40 °C, 43 °C, or 45 °C for 48 h in a series of pilot experiments. After the treatment, the pupae were transferred back to the original laboratory conditions. Soon after eclosion, adult individuals were frozen to minimize wing damage. The cold shock and heat shock conditions for subsequent experiments were determined in reference to the normality rate (the percentage of individuals that eclosed normally regardless of wing color pattern modifications) obtained in the pilot experiments.

### 2.6. Injections of Tungstate and FB28

An aqueous solution (1.0 M) of sodium tungstate (Sigma-Aldrich, St. Louis, MO, USA) or an aqueous solution (30%) of FB28 (fluorescent brightener 28 disodium salt) (Sigma-Aldrich, St. Louis, MO, USA) was injected into the abdomen of pupae within 12 h post-pupation. The injection volume was made variable for sodium tungstate (see Section 3) but was 2.0 μL for FB28. The pupae were removed from the wall of the container before injection, and injection was performed using an Ito (Terumo) microsyringe (MS-25) (Fuji, Shizuoka, Japan). After injection, they were placed back to the original position on the wall using adhesive tape.

### 2.7. Evaluation of Modifications

The following points were examined to judge whether a given individual was modified: (1) white spot size (reduction or enlargement), (2) reddish spot size (reduction or enlargement), (3) reddish spot elongation, (4) reddish spot darkness, (5) novel spot emergence, (6) increase in the number of blue scales as a sparse pattern, and (7) increase in the number of yellow scales as a sparse pattern.

To evaluate the reddish spot darkness semiquantitatively, modification scores (Score 1 to Score 4) were assigned to the most anterior spot (SRS7) of treated and nontreated individuals based on the darkness of the reddish spots by visual inspection (Figure 2c). The least reddish (beige) one was assigned Score 1, the pale reddish one was assigned Score 2, the more reddish one was assigned Score 3, and the most reddish one was assigned Score 4.

Individuals that eclosed with wrinkled wings or that could not eclose (confinement within the pupal case) were not considered cases of normal eclosion. Individuals that died of parasitic insects were excluded from the number of treated individuals. Individuals with one or more modifications that were clearly different from nontreated individuals were counted to determine the number of modified individuals. The modification rate was obtained according to the following calculation: the number of modified individuals divided by the number of adult individuals that eclosed successfully. Spot size reduction was defined as a size smaller than “mean – 2 SD (standard deviation)” of the size of the no-treatment group, and spot size enlargement was defined as a size larger than “mean + 2 SD” of the size of the no-treatment group.

### 2.8. Image Acquisition and Processing

Wings were detached by scissors from the thorax and scanned with an EPSON scanner GT-F500 (Tokyo, Japan). The right wings were used unless they were damaged. To evaluate an increase or decrease in the size of the white spots (MWS1–MWS7) and the reddish spots (SRS1–SRS7), ImageJ (bundled with 64-bit Java 1.8.01_112; https://imagej.nih.gov, accessed on 10 December 2021) was used. A wing image area was specified manually using a polygon selections tool. Alternatively, an image was converted to a 16-bit black and white image, the minimum threshold was set at 0, and the maximum threshold was set at 225 for males and nonmimetic females and at 215 for mimetic females. The size was set at 0.30–infinity (mm^2^) in “Analyze particles” to measure the whole wing area. To measure the white and reddish spot areas, the minimum threshold was set at 50. Using the area values obtained above, we obtained the white spot size and the reddish spot size with respect to the whole wing area. These values were expressed as percentages. Scale-level images were obtained using a Keyence Digital Microscope VHX-7000 (Osaka, Japan).

### 2.9. Statistical Analyses

We performed Student’s *t*-tests using Microsoft Excel (Office 365) for the reddish spot size (area value) and the white spot size (area value) with respect to the whole wing area between the treatment and no-treatment groups. Due to multiple comparisons, *p*-values were adjusted by the Bonferroni method. For score distributions, Fisher’s exact test was performed using Python 3.9.4. Distributions of the numbers of modified and nonmodified individuals were compared between the treatment and no-treatment groups. In rare cases, no SRS7 was found, in which case such individuals were excluded from the analysis. Mimetic females were not subjected to this analysis because of the high variability of the reddish spots even among a given sibling group. 

## 3. Results

### 3.1. Pilot Experiments for Cold Shock and Heat Shock Conditions

**Figure 3:**
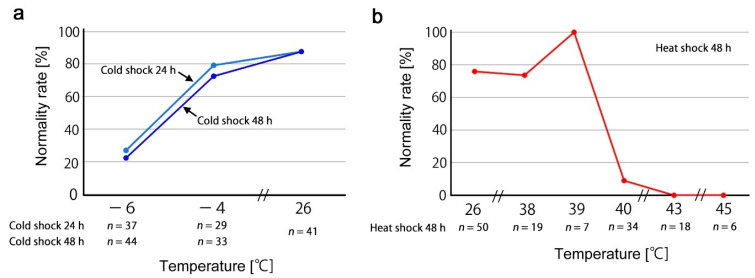
Normality rates after temperature shock treatments to determine optimal experimental temperature conditions. (**a**) Cold shock for 24 and 48 h at various temperatures. (**b**) Heat shock for 48 h at various temperatures.

To determine operational temperatures for experimental treatments that effectively induce color pattern modifications, we first examined a response profile to cold shock temperatures as a pilot experiment (Figure 3a). We examined the normality rate (the number of individuals that eclosed normally regardless of color pattern modifications). The normality rate of the −6 °C treatment was much smaller than that of the −4 °C treatment, indicating that the −6 °C treatment was too severe for this butterfly to survive. In contrast, the cold shock duration was set at either 24 h or 48 h, and these two durations did not differ much, but the latter was slightly more severe. Therefore, we decided to use the treatment conditions of −4 °C for 48 h for subsequent cold shock experiments.

We then examined a response profile to heat shock temperatures as another pilot experiment (Figure 3b). Temperatures from 26 °C to 39 °C for 48 h showed high normality rates, but the treatment temperature at 40 °C showed a dramatic decrease in normality rate. Therefore, we used the treatment conditions of 39 °C for 48 h for subsequent heat shock experiments.

### 3.2. Modifications Induced by Cold Shock

#### 3.2.1. Modification Patterns

**Figure 4:**
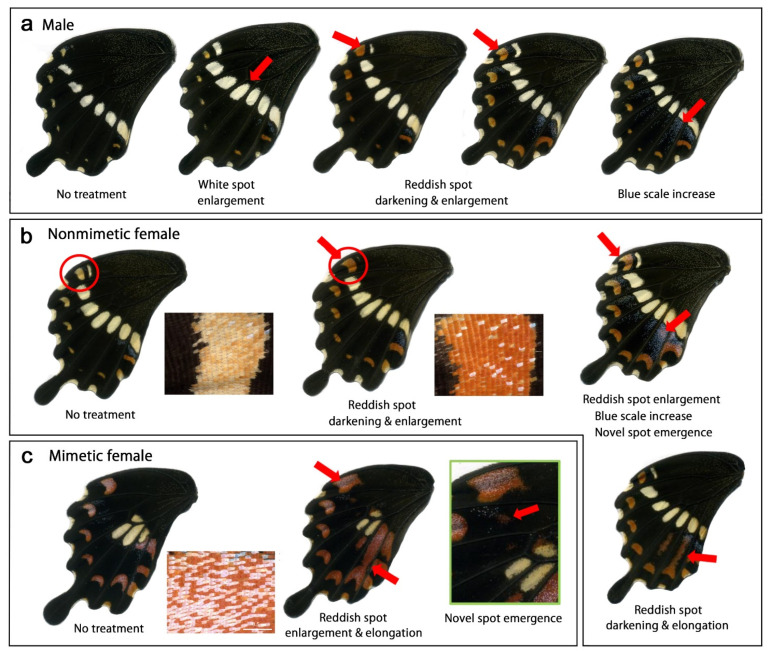
Modifications induced by cold shock in *P. polytes*. Red arrows indicate modified patterns. In many individuals, modifications are found wing-wide, but only representative patterns are indicated by arrows. (**a**) Male. (**b**) Nonmimetic Female. Insets are enlargements of SRS7 (circled). The novel reddish spot is superimposed by the blue scales. (**c**) Mimetic female. An inset of the nontreated individual is an enlargement of SRS7. The rightmost panel is an enlargement of the medial portion of the treated individual.

We performed cold shock treatment at −4 °C for 48 h (Appendix A, Table A2) as determined in the pilot experiments. In this case, normality rates for no treatment and cold shock treatment in all samples were 91.6% (*n* = 154) and 39.7% (*n* = 247), respectively, suggesting that treated pupae experienced a risk of survival. In males, we observed the following color pattern modifications (Figure 4a, Table 1): enlargement of the white spots (*n* = 5), enlargement of the reddish spots (*n* = 25), darkening of the reddish spots (*n* = 29), and increase in the blue scales (*n* = 2). The modification rate was 87.0% (*n* = 46) in the treatment group and 3.3% (*n* = 61) in the no treatment control group, indicating that the treatment was successfully performed.

In nonmimetic females, we observed the following color pattern modifications (Figure 4b, Table 1): enlargement of the white spots (*n* = 5), enlargement of the reddish spots (*n* = 8), darkening of the reddish spots (*n* = 28), and increase in the blue scales (*n* = 4). In addition, we observed the emergence of medial reddish spots (*n* = 4) (Figure 4b). This modification was not observed in males. Medial reddish spots are not present in nonmimetic females but are present in mimetic females. Furthermore, the submarginal reddish spots were elongated toward the proximal side (Figure 4b). The modification rate was 96.9% (*n* = 32) in the treatment group and 17.9% (*n* = 39) in the no-treatment control group.

In mimetic females, we observed the following color pattern modifications (Figure 4c, Table 1): enlargement of the white spots (*n* = 2), enlargement of the submarginal reddish spots (*n* = 3), and enlargement of the medial reddish spots (*n* = 1). This latter individual also had novel medial reddish spots at the anterior compartments (Figure 4c). The modification rate was 37.5% (*n* = 16) in the treatment group and 0% (*n* = 21) in the no-treatment control group.

#### 3.2.2. Quantitative Evaluation

**Figure 5:**
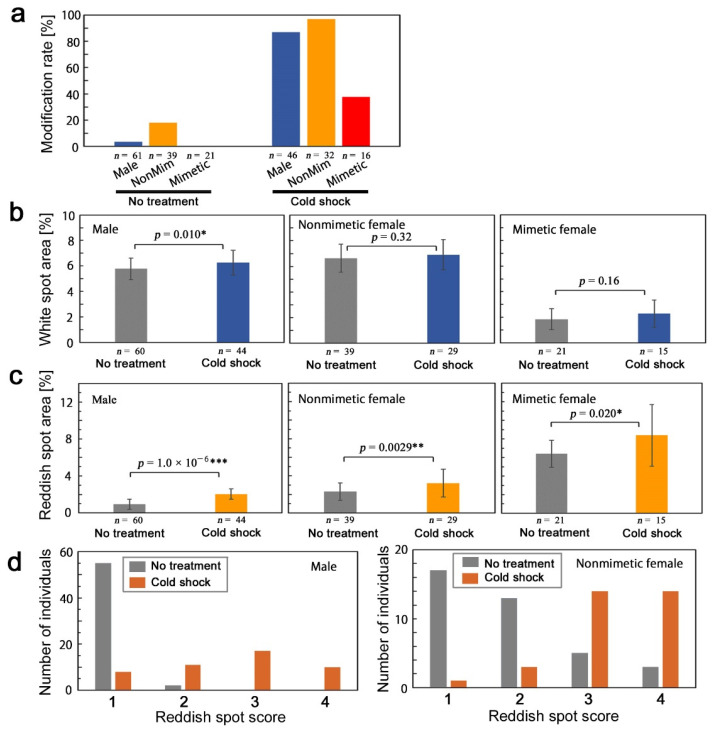
Quantitative evaluations of cold-shock-induced modifications in *P. polytes*. Asterisks indicate levels of statistical significance; * *p* < 0.05; ** *p* < 0.01; *** *p* < 0.001. (**a**) Modification rates among males, nonmimetic females, and mimetic females. (**b**) White spot area. (**c**) Reddish spot area. (**d**) Distributions of reddish spot scores.

We compared modification rates among males, nonmimetic females, and mimetic females (Figure 5a). Nonmimetic females showed the largest modification rate in response to cold shock treatment, although nonmimetic females also had a relatively high “modification” rate with no treatment. The modification rate of males was slightly lower than that of nonmimetic females. Mimetic females showed the lowest modification rate.

The white spot (MWS1–MWS7) size (the white spot area value divided by the whole wing area value) was compared between the treatment and no-treatment groups (Figure 5b). In males, the white spot size was significantly larger in the treatment group than in the no-treatment group (*p* = 0.010). In nonmimetic females, there was no significant difference between the treatment group and no-treatment group (*p* = 0.32), although some individuals showed enlargement. In mimetic females, there was no significant difference (*p* = 0.16).

Similarly, the submarginal reddish spot (SRS1–SRS7) size (the reddish spot area value divided by the whole wing area value) was compared (Figure 5c). The reddish spots were enlarged significantly in males (*p* = 1.0 × 10^–6^), mimetic females (*p* = 0.0029), and mimetic females (*p* = 0.020), although these *p*-values increased in this order.

The SRS scores were evaluated here (Figure 5d). In males with treatment (*n* = 46), we observed Score 1 (*n* = 8), Score 2 (*n* = 11), Score 3 (*n* = 17), and Score 4 (*n* = 10). In contrast, without treatment (*n* = 57), Score 1 was the majority (*n* = 55), Score 2 was the minority (*n* = 2), and Score 3 and Score 4 were not observed. Here, Score 1 was considered not modified (no change), and other scores were considered modified. The distributions of these two groups were significantly different (*p* = 6.9 × 10^−18^, Fisher’s exact test), suggesting that the darkening of the reddish spots was induced by the cold shock treatment. 

In nonmimetic females with treatment (*n* = 32), we observed Score 1 (*n* = 1), Score 2 (*n* = 3), Score 3 (*n* = 14), and Score 4 (*n* = 14). In contrast, without treatment (*n* = 38), we observed Score 1 (*n* = 17), Score 2 (*n* = 13), Score 3 (*n* = 5), and Score 4 (*n* = 3). Here, Score 1 and Score 2 were considered not modified (no change), and Score 3 and Score 4 were considered modified. The distributions of these two groups were significantly different (*p* = 1.8 × 10^−8^, Fisher’s exact test), suggesting again that the darkening of the reddish spots was induced by the cold shock treatment.

### 3.3. Modifications Induced by Heat Shock

#### 3.3.1. Modification Patterns

**Figure 6:**
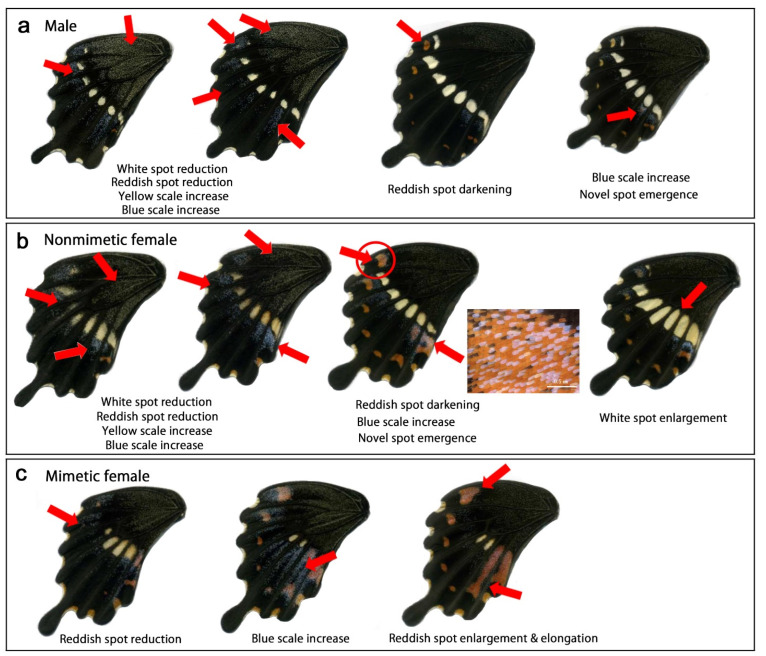
Modifications induced by heat shock in *P. polytes*. Red arrows indicate modified patterns. In many individuals, modifications were found wing-wide, but only representative modified patterns are indicated by arrows. For the color patterns of the no-treatment groups, see Figure 4. (**a**) Male. (**b**) Nonmimetic female. An inset is an enlargement of SRS7 (circled). (**c**) Mimetic female.

We performed heat shock treatment at 39 °C for 48 h (Appendix A, Table A3) as determined in the pilot experiments. In this case, normality rates for no treatment and heat shock treatment were 75.5% (*n* = 110) and 43.1% (*n* = 195), respectively, suggesting that treated pupae experienced a risk of survival. In males, we observed the following color pattern modifications (Figure 6a, Table 1): reduction of white spots (*n* = 7), reduction of reddish spots (*n* = 3), and darkening of reddish spots (*n* = 15). In addition, an increase in yellow scales at the basal area (*n* = 5) was observed. Furthermore, although minor, an increase in medial blue scales (*n* = 2) and emergence of novel medial spots (*n* = 2), enlargement of white spots (*n* = 1), and enlargement of reddish spots (*n* = 2) were also observed. Both enlargement and reduction were observed after the same heat shock treatment, and this aspect of the heat shock treatment differed from that of the cold shock treatment. The modification rate was 70.6% (*n* = 41) in the treatment group and 0% (*n* = 34) in the no-treatment group.

Similarly, in the nonmimetic females (Figure 6b, Table 1), we observed a reduction in white spots (*n* = 9), darkening of reddish spots (*n* = 15), and an increase in medial blue scales (*n* = 5). In addition, although minor, we observed enlargement of the white spot (*n* = 1), reduction of reddish spots (*n* = 2), enlargement of reddish spots (*n* = 1), and an increase in yellow scales in the basal area (*n* = 1). The modification rate was 92.6% (*n* = 27) in the treatment group and 40.1% (*n* = 27) in the no-treatment group.

In the mimetic females (Figure 6c, Table 1), we observed reduction of white spots (*n* = 1), enlargement of the white spot (*n* = 2), reduction of reddish spots (*n* = 6), enlargement of reddish spot (*n* = 4), an increase in medial blue scales (*n* = 3), and an increase in yellow scales in the basal area (*n* = 2). The darkening of reddish spots was not differentiated due to high natural variations even in the no-treatment group. The modification rate was 55.0% (*n* = 20) in the treatment group and 0% (*n* = 16) in the no-treatment group.

#### 3.3.2. Quantitative Evaluation

**Figure 7:**
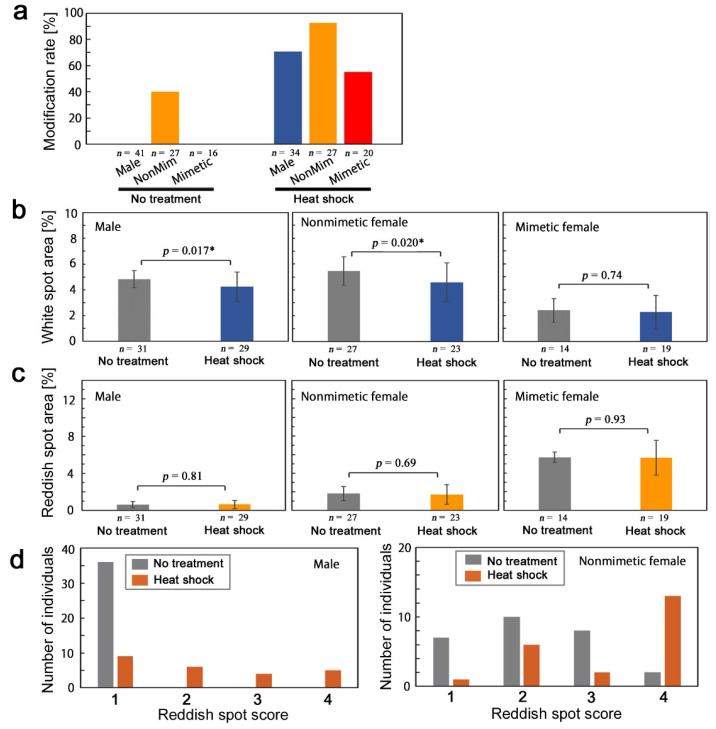
Quantitative evaluations of the heat-shock-induced modifications in *P. polytes*. Asterisks indicate statistical significance; * *p* < 0.05. (**a**) Modification rates among males, nonmimetic females, and mimetic females. (**b**) White spot area. (**c**) Reddish spot area. (**d**) Distributions of reddish spot scores.

We compared modification rates among males, nonmimetic females, and mimetic females (Figure 7a). Nonmimetic females showed the largest modification rate in response to the heat shock treatment, although nonmimetic females also had a relatively high “modification” rate with no treatment. The modification rate of males was lower than that of nonmimetic females. Mimetic females showed the lowest modification rate. These results were consistent with those of the cold shock treatment (Figure 5a).

Here, we examined white spot (MWS1–MWS7) size (white spot area value divided by the whole wing area value) and reddish spot (SRS1–SRS7) size (reddish spot area value divided by the whole wing area value). White spot size was significantly smaller in the treatment group than in the no-treatment group in males (*p* = 0.017, *t*-test) and in nonmimetic females (*p* = 0.020, *t*-test), but this difference was not observed in mimetic females (Figure 7b). On the other hand, the reduction of reddish spot size was not significant in males (*p* = 0.81, *t*-test), in nonmimetic females (*p* = 0.69, *t*-test), and in mimetic females (*p* = 0.93, *t*-test) (Figure 7c).

The SRS scores were evaluated here (Figure 7d). In males, in the no-treatment group (*n* = 36), all individuals scored 1 (*n* = 36). In the treatment group (*n* = 24), we observed scores of 1 (*n* = 9), 2 (*n* = 6), 3 (*n* = 4), and 4 (*n* = 5). A score of 1 was considered not modified (no change), and scores of 2, 3, and 4 were considered modified. The distributions of these two groups were significantly different (*p* = 2.5 × 10^−8^, Fisher’s exact test), suggesting that the darkening of the reddish spots was induced by the heat shock treatment. In nonmimetic females, we observed scores of 1 (*n* = 7), 2 (*n* = 10), 3 (*n* = 8), and 4 (*n* = 2) in the no-treatment group (*n* = 27), and we observed scores of 1 (*n* = 1), 2 (*n* = 6), 3 (*n* = 2), and 4 (*n* = 13) in the treatment group. Scores of 1 and 2 were considered not modified (no change), and scores of 3 and 4 were considered modified. They were significantly different (*p* = 0.045, Fisher’s exact test), suggesting that the darkening of reddish spots was induced in the nonmimetic females.

#### 3.3.3. Sibling-Dependent Response

**Figure 8:**
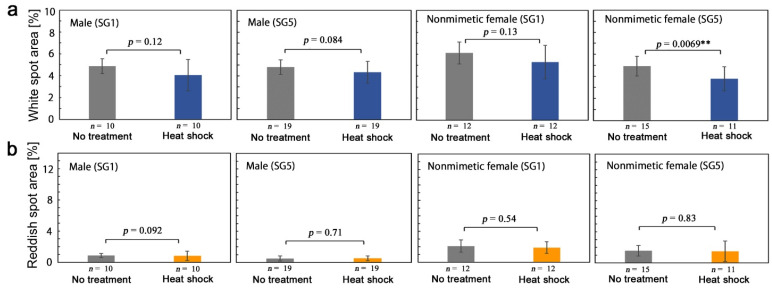
Quantitative evaluations of heat-shock-induced modifications in two sibling groups (SG1 and SG5) in *P. polytes*. Asterisks indicate statistical significance; ** *p* < 0.01. (**a**) White spot area. (**b**) Reddish spot area.

For the heat shock treatment above, we used two sibling groups (SG1 and SG5) for males and nonmimetic females. Here, we examined modification responses within a single sibling group, SG1 or SG8, and compared their results (Appendix A, Table A4). White spot size (area value) did not show a significant difference in SG1 and SG5 males or in SG1 nonmimetic females but showed a significant difference in SG5 nonmimetic females (Figure 8a). Reddish spot size (area value) did not show any significant difference in SG1 and SG5 (Figure 8b). These results suggest that a sibling dependence (genetic dependence) of modification induction was present at least in white spot size.

### 3.4. Modification Inducers: Tungstate and FB28

**Figure 9:**
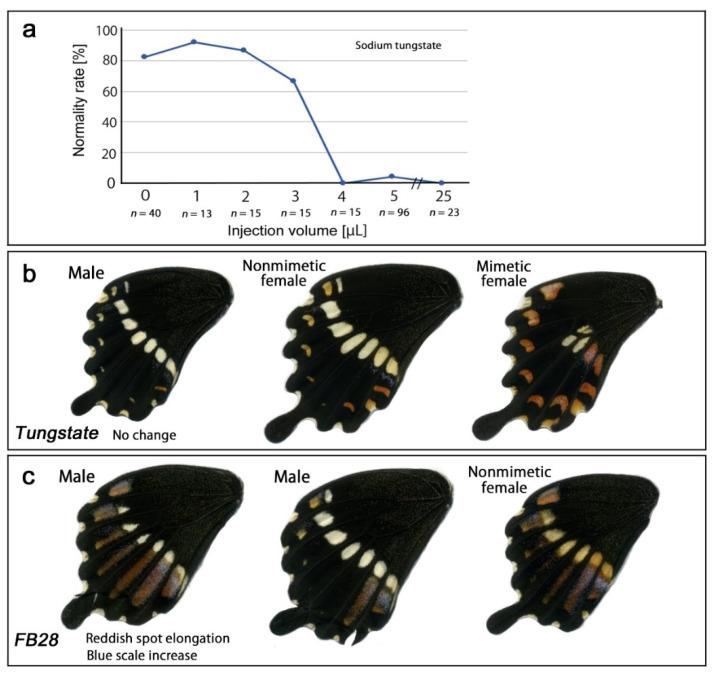
Pharmacological treatments of *P. polytes*. For the color patterns of the no-treatment groups, see Figure 6. (**a**) Normality rate in response to injection volume of a sodium tungstate solution (1.0 M) in *P. polytes*. (**b**) Injection of sodium tungstate (1.0 M, 2.0 μL). No modification was observed. (**c**) Injection of FB28 (30%, 2 μL). Distinct inward elongation of SRSs was observed.

We injected a sodium tungstate solution (1.0 M) with various injection volumes (1.0–5.0 and 25.0 μL). An increase in injection volume simply decreased the normality rate without modifications (Figure 9a). No modification was observed (Figure 9b, Table 1). We also injected FB28, a novel modification inducer found in a series of injection experiments, into the blue pansy butterfly *Junonia orithya*, where FB28 induced TS-type modifications similar to those from cold shock and tungstate [56]. In the present study, FB28 induced distinct inward elongation of reddish spots (Figure 9c, Table 1), which was similar to one of the modifications induced by the cold shock treatment (Figure 4b) or heat shock treatment (Figure 6c). In total, normality rates for no treatment and FB28 treatment were 93.6% (*n* = 47) and 84.6% (*n* = 13), respectively.

### 3.5. Summary of Modifications

The results of the present study are summarized in Table 1. Both white and reddish spot areas tended to be enlarged by cold shock and reduced by heat shock, but both treatments made reddish spots darker. Reddish spot darkening and enlargement induced by cold shock in males and nonmimetic females are reminiscent of the natural color patterns of mimetic females, suggesting a contribution of phenotypic plasticity to mimetic color pattern evolution. Interestingly, the inward elongation of the submarginal reddish spots was induced not only by temperature shock treatments but also by FB28 treatment.

## 4. Discussion

### 4.1. Modification Induction in Papilionid Butterflies and P. polytes

In this study, we successfully obtained physiologically induced color pattern modifications in the common Mormon butterfly *P. polytes*. Some *Papilio* butterflies have already been subjected to chemical injections [25,27,29,30]. In one study, *Papilio xuthus* was subjected to injections of acid carboxypeptidase [25]. Injections of sodium tungstate were made in two papilionid butterflies, *P. xuthus* and *Graphium sarpedon*, but without modifications [27], which is consistent with the present study and suggests mechanistic differences in developmental color pattern formation from nymphalid and lycaenid butterflies. In contrast, the high sensitivity of *P. polytes* to FB28 was unexpected. Cold-shock hormones (CSHs) [34] or their receptors may be different among families of butterflies.

Despite these pharmacological experiments, to our knowledge, the present study is the first systematic quantitative report on the successful induction of color pattern modifications by temperature shock treatments in papilionid butterflies. The use of temperature shock may not be as robust as chemical inducers but is more relevant in development and evolution in the real world; temperature differences are likely an important inducer of phenotypic plasticity in the field. A recent qualitative report on cold shock results using *Pterourus (Papilio) glaucus* [61] is interesting, but quantitative analyses are necessary to accurately evaluate effects of cold shock treatment.

In the heat shock treatment, modifications varied more than those in the cold shock treatment, as expected from previous studies in *J. orithya* [37] and *Vanessa indica* [39]. We observed modification differences between two sibling groups, SG1 and SG8, indicating lineage dependence (hence genetic dependence) of modifications. This result suggests the importance of genetic and/or maternal effects for the evolution of mimetic patterns. Indeed, the lineage-dependence of lepidopteran wing color patterns is known [14,62]. Nevertheless, overall, the heat-shock response may be considered in an opposite direction from the cold-shock response based on the reduction of the central white spots and the reddish submarginal spots in all sexes and forms. Importantly, these modifications in males and nonmimetic females were similar to the natural color patterns of mimetic females.

In *P. polytes*, males, nonmimetic females, and mimetic females all showed modifications but at different rates. Nonmimetic females showed the highest modification rate, and mimetic females showed the lowest modification rate in both the cold shock and heat shock treatments, indicating a higher potential for plasticity in nonmimetic females, which is reasonable if one considers that the mimetic form is a result of genetic assimilation of modification-sensitive genotypes. Considering that males and nonmimetic females show similar color patterns but very different modification rates, this sensitivity difference indicates sex-biased mechanisms of phenotypic plasticity. Moreover, the lowest sensitivity of the mimetic form strongly suggests that the mimetic form may be an end-product of plasticity-based evolution. Further studies would be needed to demonstrate this process.

In the fruit fly *Drosophila melanogaster*, it appears that heat-shock proteins serve as a reservoir of mutations and that heat shock treatment beyond the function of heat-shock proteins causes phenotypic expression of various aberrations [63]. Because injection of geldanamycin, a heat-shock-protein inhibitor, does not cause highly variable phenotypes in *J. orithya*, heat-shock proteins may not play a major role in color pattern modifications [18], but further investigation in this line may be necessary.

### 4.2. Real-Time Evolution via Phenotypic Plasticity

Most butterfly color patterns are considered evolutionary consequences of “modifications” of the nymphalid groundplan [9,57,64,65,66,67]. In this sense, experimentally induced modifications are often indicative of evolutionary history and mechanisms. The present study showed that the color pattern evolution mechanisms postulated in nonmimetic nymphalid, lycaenid, and pierid butterflies appeared to be applicable to mimicry evolution. Natural selection by birds [68] and sympatric abundance of model species [69,70,71,72] are important in mimicry evolution in *P. polytes*, but a pallet of phenotypes from which some can be selected for fitness may be provided by phenotypic plasticity in response to environmental stress not only in nonmimicry color pattern evolution but also in mimicry evolution.

For modified color patterns to be assimilated in a population, modifications should be functionally relevant. A gene responsible for a given plastic phenotype may be epistatically linked with different genes that have a survival advantage, as suggested in the case of *Vanessa* butterflies [38,39,40,41] and *Zizeeria maha* [43]. In the case of *P. polytes*, modifications can be readily functional if they are similar to the color patterns of unpalatable species. It is reasonable to conclude that the present study added a potential example of real-time evolution (genetic assimilation) of color patterns based on phenotypic plasticity.

However, in the case of *P. polytes*, not all differences between nonmimetic and mimetic color patterns were induced by physiological treatments. The central white spot (CWS) was not induced in this study. Likewise, enlargement of medial white spots in middle compartments together with reduction in other compartments were not achieved. These results demonstrate that the mimetic color pattern evolution of *P. polytes* may be more complex than the color pattern evolution of nonmimetic species such as *Z. maha* and the genus *Vanessa.*

**Figure 10:**
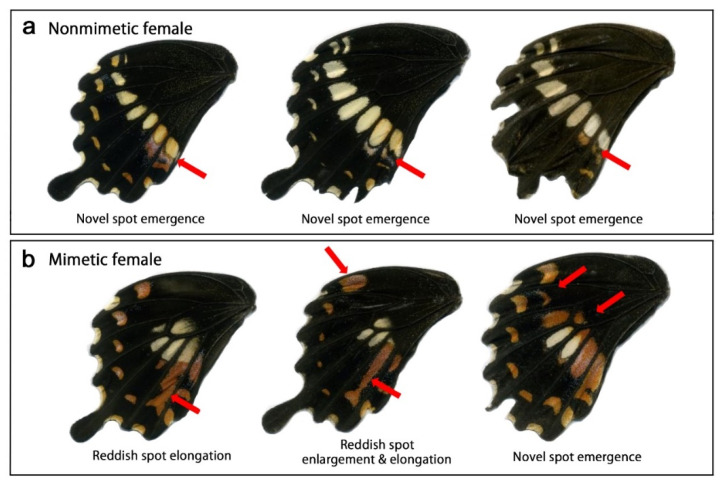
Various color patterns of field-caught female individuals of *P. polytes* on Okinwa-jima Island in 2019–2021. Red arrows indicate naturally modified patterns. (**a**) Nonmimetic female. Novel spots, which may correspond to MRS1 and MRS2 in the mimetic female, are present in these individuals. (**b**) Mimetic female. Enlargement and elongation of the reddish spots and novel spot emergence are observed.

The Ryukyu Archipelago (Okinawa) is the northern range margin of *P. polytes*, a local population that may be relatively easily affected by evolutionary pressure. Highly variable field-caught specimens of *P. polytes* collected in Okinawa during the period of 2019–2021 (Figure 10) suggest that *P. polytes* color patterns are potentially responding to environmental stress in the field even now. In other words, the color pattern evolution of *P. polytes* may be ongoing in Okinawa, and the process of genetic assimilation has not yet been completed. Sympatric dimorphism of *P. polytes* in Okinawa may be an indication of real-time color-pattern evolution and incomplete genetic assimilation, an equilibrium of which could shift in response to the abundance of the model individuals and predation pressure from birds.

The above interpretation is reasonable based on the following studies. Historically, on Okinawa-jima Island, the model species *P. aristolochiae* was found in 1993 [69]. Until that time, the medial white spots in the mimetic females of *P. polytes* were relatively small, but after the arrival of the model species, the medial white spots increased in size [70], suggesting that predation pressure by birds drives natural selection for the mimetic color patterns. The mimicry rates of *P. polytes* on islands are determined by the density of individuals of the model species [69,70,71,72], which has been supported by genetic analyses [73] and population dynamics’ models [74].

It has been reported that reddish spots change in size during the summer from May to August in Okinawa [75]. This change has been attributed to ultraviolet (UV) radiation [75] but may also be induced by natural heat stress. It has been reported that when field-caught females were irradiated with UV, their offspring showed color pattern modifications, although regrettably, such wing images have not been presented [75]. At present, it is reasonable to conclude that temperature shock is an important environmental stressor that could drive evolution in butterflies, including *P. polytes*, based on the present study, but a potential contribution of UV stress should also be investigated in the future.

In the same study [75], UV-induced melaninization was interpreted as a trade-off between two adaptive states: a mimetic phenotype for protection against predators and a melaninization phenotype for wing protection against UV irradiation. This trade-off hypothesis is interesting but needs further thought because melaninization (i.e., reduction of the white or reddish spots in size or general darkening) can be induced nonspecifically by cold shock, heat shock, modification inducers, and general-stress inducers in a variety of butterflies and moths, which are nonadaptive phenotypic modulation. Melaninization is, therefore, unlikely to be adaptive because it is not specific to UV irradiation or this species. However, there is a possibility that this nonadaptive plasticity may be evolving to be adaptive in Okinawa.

Experimental treatments of butterflies for revealing their phenotypic plasticity do not have to be similar to the environmental stress that their ancestors might have possibly experienced. In this sense, the results of pharmacological treatments are relevant as long as information on phenotypic plasticity can be obtained, assuming that the present species retain phenotypic plasticity of their ancestors at least to some extent. Nonetheless, it is tempting to ask what kind of environmental stress the ancestors might have received. Butterfly species that naturally show a resemblance of TS-type modifications of other related species are often distributed in high-altitude or high-latitude areas where the temperature difference within a day may be high [27,38,39,40,41,42,43]. These cases suggest that natural cold shock might have played a role in color pattern evolution.

Populations in the Ryukyu Archipelago (Okinawa) are considered the northern range margin populations of *P. polytes*, but there is no high-altitude area in these subtropical islands. Indeed, air temperatures in Okinawa cannot reach the cold shock and heat shock conditions used in this study (−4 and 39 °C, respectively). However, we believe that there are reasonable occasions in the field where pupae are subjected to serious temperature stress or “natural temperature shock”, even in temperate and subtropical regions. Temperature stress may be more serious when pupae receive direct sunshine that raises pupal body temperatures quickly even in winter. In an opposite manner, pupae may be soaked in water after a local flood, which chills pupae effectively in winter. In these cases, pupae should be accidentally moved to a life-threatening microenvironment by natural or artificial disasters. We speculate that such temperature shocks to individual pupae above or below air temperatures are not very rare in the field, even in subtropical regions such as Okinawa. To generalize, a natural temperature shock to pupae may be a driving factor for butterfly and other insect diversity via phenotypic plasticity. This hypothesis should be tested through various field and laboratory experiments in the future.

### 4.3. Evolution of Mimetic and Nonmimetic Female-Limited Dimorphism

In many butterflies, only females show mimicry, as seen in *P. polytes*. The evolution of female-limited mimetic color patterns in papilionid butterflies has been enigmatic since Darwin and Wallace. Genes responsible for this color pattern diversity in *P. polytes* have been identified [50,51,52,53,54,55], but identifying genes for color patterns may not be sufficient to explain the sex dependence.

It is important to consider that female-limited dimorphism or polymorphism is not rare in nonmimetic butterflies. Examples from Japanese butterflies are as follows [17]. The pierid butterfly *C. erate* shows white and yellow forms in females, but there is only a yellow form in males. The lycaenid butterfly, *Chrysozephyrus brillantinus*, has four forms (A, B, AB, and O types) in females, but there is only a single form in males that is different from any other female form. In the nymphalid butterfly *J. orithya*, females show various color patterns, including orange and blue forms, but males show only a single blue form. Dimorphic mimetic color patterns in *P. polytes* may be considered a case of female-limited color pattern diversity that is widely seen in butterflies.

Explanations for female-limited mimicry are multiple. Females are often under higher predation pressure than males due to their higher nutritious quality of eggs in the abdomen [44,45]. The mimetic form has some disadvantages; it is less active [47,48] and has shorter life expectancy [49]. The mimetic forms are not always effective; their effectiveness depends on the number of sympatric individuals of the model species [69,70,71,72,73,74].

The present study adds a new important perspective that is not mutually exclusive to the explanations above. Phenotypic plasticity that provides a pallet of phenotypes for natural selection is larger in females than in males. This possibility is supported by the fact that the lycaenid butterfly *Z. maha* shows female-biased modifications [43], although this species is not related to mimicry at all. Similarly, the wing color of females is more variable than that of males in *Pieris napi*, a nonmimetic species [76].

## 5. Conclusions

The common Mormon butterfly *P. polytes* exhibited unique wing color pattern modifications as phenotypic plasticity (phenotypic modulation) in response to cold shock and heat shock treatments and to FB28 injections but not to tungstate injections, suggesting similarities to and differences from lycaenid and nymphalid butterflies. Modifications induced in males and nonmimetic females at least partly resembled the natural color patterns of the mimetic females, suggesting that the mimetic color patterns in *P. polytes* might have evolved from plastic nonmimetic phenotypes in response to environmental stress through a genetic assimilation process. Nonmimetic females had a higher modification rate than males and mimetic females did, similar to the case of the nonmimetic lycaenid species *Zizeeria maha*. Female-limited color patterns that are widely seen in butterflies (including nonmimetic ones) may be related to sex-biased modification rates in butterflies. The present study sheds light on the development and evolution of female-limited mimetic wing color patterns not only in *P. polytes* but also in other butterflies.

## Figures and Tables

**Table 1:** Summary of wing color pattern modifications induced by experimental treatments.

Treatment Mode	White Spot Area	Reddish Spot Area	Reddish Spot Darkness	Novel Spot
Cold shock	Enlarged in males	Enlarged, Elongated	Darkened	Emerged only in females
Heat shock	Reduced	Reduced (+Enlarged), Elongated	Darkened	Emerged in both sexes
Sodium tungstate	No change	No change	No change	No change
FB28	Not applicable	Elongated	Not applicable	No change

Note: In the FB28 treatment, both white and reddish spots were obscured by the elongated reddish spots, and the quantification of area values was not performed (not applicable).

## Data Availability

The data presented in this study are available in this article and in Appendix A. Additional data that support the findings of this study are available from the authors upon request.

## Appendix A

Here, we present tables regarding sibling groups used in this study (Table A1) and the number of individuals that had modifications after a treatment (Table A2, Table A3 and Table A4).

**Table A1:** Sibling groups (SGs) used in this study.

Sibling Group	Mother	Treatment
SG1	Nonmimetic	Heat shock and cold shock
SG2	Mimetic	Cold shock
SG3	Nonmimetic	Cold shock
SG4	Nonmimetic	Cold shock
SG5	Nonmimetic	Heat shock
SG6	Mimetic	Tungstate injection (1 M, 5 or 25 μL)
SG7	Nonmimetic	Tungstate injection (1 M, 1–4 μL)
SG8	Nonmimetic	FB28 injection (30%, 2 μL)

Note: Groups used for pilot studies and other trials without success are not included in this table. The heat shock and cold shock conditions were performed at 39 °C for 48 h and at −4 °C for 48 h, respectively.

**Table A2:** Number of modified individuals after the cold shock treatment.

Modifications	Male		Nonmimetic Female		Mimetic Female	
	NT	CS	NT	CS	NT	CS
Total number of individuals	61	46	39	32	21	16
White spot reduction	0	0	0	0	0	0
White spot enlargement	0	5	0	5	0	2
Reddish spot reduction	0	0	0	0	0	0
Reddish spot enlargement	0	25	0	8	0	3
Reddish spot darkening	2	29	7	28	0	0
Reddish spot elongation	0	0	0	1	0	0
Novel spot emergence	0	0	0	4	0	1
Increase in blue scales	0	2	0	4	0	0
Increase in yellow scales (forewing)	0	0	0	0	0	0
Increase in yellow scales (hindwing)	0	0	0	0	0	0
Number of modified individuals	2	40	7	31	0	6
Modification rate (%)	3.3%	87.0%	17.9%	96.9%	0%	37.5%

Note: NT and CS indicate no treatment and cold shock treatment, respectively.

**Table A3:** Number of modified individuals after the heat shock treatment.

Modifications	Male		Nonmimetic Female		Mimetic Female	
	NT	HS	NT	HS	NT	HS
Total number of individuals	41	34	27	27	16	20
White spot reduction	0	7	0	6	0	1
White spot enlargement	0	1	0	1	0	2
Reddish spot reduction	0	3	0	1	0	6
Reddish spot enlargement	0	2	0	1	0	4
Reddish spot darkening	0	15	10	15	0	0
Reddish spot elongation	0	0	0	0	0	0
Novel spot emergence	0	2	1	11	0	0
Increase in blue scales	0	2	0	5	0	3
Increase in yellow scales (forewing)	0	3	0	4	0	0
Increase in yellow scales (hindwing)	0	5	0	1	0	2
Number of modified individuals	0	24	11	25	0	11
Modification rate (%)	0%	70.6%	40.1%	92.6%	0%	55.0%

Note: NT and HS indicate no treatment and heat shock treatment, respectively.

**Table A4:** Number of modified individuals in sibling groups after the heat shock treatment.

Modifications	Male				Nonmimetic Female			
	SG1		SG5		SG1		SG5	
	NT	HS	NT	HS	NT	HS	NT	HS
Total number of individuals	10	10	31	24	12	12	15	15
White spot reduction	0	4	0	3	0	3	0	3
White spot enlargement	0	1	0	0	0	1	0	0
Reddish spot reduction	0	3	0	0	0	0	0	1
Reddish spot enlargement	0	2	0	0	0	0	0	1
Reddish spot darkening	0	3	0	12	3	0	7	7
Reddish spot elongation	0	0	0	0	0	8	0	0
Novel spot emergence	0	0	0	2	0	3	1	8
Increase in blue scales	0	1	0	1	0	3	0	2
Increase in yellow scales (forewing)	0	0	0	3	0	1	0	3
Increase in yellow scales (hindwing)	0	1	0	4	0	0	0	1
Number of modified individuals	0	8	0	16	3	11	8	14
Modification rate (%)	0%	80.0%	0%	66.7%	25.0%	91.7%	53.3%	93.3%

Note: NT and HS indicate no treatment and heat shock treatment, respectively.
